# Supplementary material for: Better sleep, better life? testing the role of sleep on quality of life
Source: PLoS One. 2023 Mar 15;18(3):e0282085. doi: 10.1371/journal.pone.0282085 (PMC10016705; doi:10.1371/journal.pone.0282085)
Supplement: S1 Table — Note: *** p<0.001, ** p<0.01, * p<0.05, 95% CI in parentheses. (PDF) [file pone.0282085.s002.pdf]

S2 Table 1. Null models of sleeping habits and quality of life, linear mixed models with repeated measurements.

	**Life satisfaction**		**Wellbeing**		**Subjective health**		**Work stress**		**Happiness**	
Constant	7.43***	(7.36 - 7.49)	4.07***	(4.04 - 4.11)	3.07***	(3.03 - 3.11)	.19***	(.18 - .20)	7.31***	(7.24 - 7.38)
Observations	4,523		3,850		3,867		2,097		3,857	
Households	2,155		2,100		2,105		1,305		2,101	
AIC	17714		9941		10294		9		14894	
BIC	17752		9979		10332		43		14932	
ICC households	16%		9%		11%		3%		13%	
ICC individuals	73%		85%		91%		93%		86%	
ll	-8851		-4965		5141		2		-7441	

Note: *** p<0.001, ** p<0.01, * p<0.05, 95% CI in parentheses
