# Supplementary material for: Better sleep, better life? testing the role of sleep on quality of life
Source: PLoS One. 2023 Mar 15;18(3):e0282085. doi: 10.1371/journal.pone.0282085 (PMC10016705; doi:10.1371/journal.pone.0282085)
Supplement: Attachment — Submitted filename: Response to reviewers.docx [file pone.0282085.s003.docx]

Journal requirements:

When submitting your revision, we need you to address these additional requirements.

**RESPONSE:** The manuscript and the title page documents were updated to meet PLOS ONE’s style requirements. Due to the nature of the changes, they were not made via the “track change” function. After applying the formal changes to the Manuscript, this updated version was used as a baseline for the new version of “Manuscript” as well as “Revised manuscript with track changes”.

2.In your Data Availability statement, you have not specified where the minimal data set underlying the results described in your manuscript can be found. PLOS defines a study's minimal data set as the underlying data used to reach the conclusions drawn in the manuscript and any additional data required to replicate the reported study findings in their entirety. All PLOS journals require that the minimal data set be made fully available. For more information about our data policy, please see http://journals.plos.org/plosone/s/data-availability.

Upon re-submitting your revised manuscript, please upload your study’s minimal underlying data set as either Supporting Information files or to a stable, public repository and include the relevant URLs, DOIs, or accession numbers within your revised cover letter. For a list of acceptable repositories, please see http://journals.plos.org/plosone/s/data-availability#loc-recommended-repositories. Any potentially identifying patient information must be fully anonymized.

Important: If there are ethical or legal restrictions to sharing your data publicly, please explain these restrictions in detail. Please see our guidelines for more information on what we consider unacceptable restrictions to publicly sharing data: http://journals.plos.org/plosone/s/data-availability#loc-unacceptable-data-access-restrictions. Note that it is not acceptable for the authors to be the sole named individuals responsible for ensuring data access.

We will update your Data Availability statement to reflect the information you provide in your cover letter.

**RESPONSE:** Upon submission, we have provided the following statement in the Data Availability section: *“The fourth wave (2018) of the data is publicly available. The data are deposited to the public data depository (Czech Social Science Data Archive):* [*http://nesstar.soc.cas.cz/webview/index.jsp?v=2&submode=abstract&study=http%3A%2F%2F147.231.52.118%3A80%2Fobj%2FfStudy%2FCHPSEN401&mode=documentation&top=yes*](http://nesstar.soc.cas.cz/webview/index.jsp?v=2&submode=abstract&study=http%3A%2F%2F147.231.52.118%3A80%2Fobj%2FfStudy%2FCHPSEN401&mode=documentation&top=yes)*.CSDA is part of the Consortium of European Social Science Data Archives (CESSDA). It provides open access to social science data for non-commercial use. The fifth and sixth waves of CHPS (2019-2020) are strictly confidential due to the inclusion of sensitive information and thus not publicly available. However, the data could be shared upon reasonable request on an individual basis. Also, to comply with the PLOS ONE requirements, by the time of manuscript publication, we will make sure the data will be made publicly available at least to the extent of the submitted article.”*

3.We note that you have indicated that data from this study are available upon request. PLOS only allows data to be available upon request if there are legal or ethical restrictions on sharing data publicly. For more information on unacceptable data access restrictions, please see <http://journals.plos.org/plosone/s/data-availability#loc-unacceptable-data-access-restrictions>.

**RESPONSE:** Two out of three used waves contain sensitive health-related information. Therefore, we will share a minimal data set (combined for all three waves in a long format) to comply with the PLOS ONE requirements. The data set along with the syntax used for our analyses will be part of the Supporting Information.

In your revised cover letter, please address the following prompts:

a) If there are ethical or legal restrictions on sharing a de-identified data set, please explain them in detail (e.g., data contain potentially sensitive information, data are owned by a third-party organization, etc.) and who has imposed them (e.g., an ethics committee). Please also provide contact information for a data access committee, ethics committee, or other institutional body to which data requests may be sent.

b) If there are no restrictions, please upload the minimal anonymized data set necessary to replicate your study findings as either Supporting Information files or to a stable, public repository and provide us with the relevant URLs, DOIs, or accession numbers. For a list of acceptable repositories, please see <http://journals.plos.org/plosone/s/data-availability#loc-recommended-repositories>.

We will update your Data Availability statement on your behalf to reflect the information you provide.

**RESPONSE:** Data contain sensitive information (medical test results) that is not possible to share. Therefore, we provide the minimal data set that is available in a Supporting Information file along with the syntax used for our analyses.

4.Please include your full ethics statement in the ‘Methods’ section of your manuscript file. In your statement, please include the full name of the IRB or ethics committee who approved or waived your study, as well as whether or not you obtained informed written or verbal consent. If consent was waived for your study, please include this information in your statement as well.

**RESPONSE:** We provide this information in the system’s section Ethics Statement (“study approved by Ethics Committee of the Institute for Clinical and Experimental Medicine and Thomayer Hospital in Prague: study number G-16–05–02, and written consent obtained”). We have included this very same information in the manuscript as requested (page 8).

Additional Editor Comments:

This is an interesting article and has merit for publication. However, there are several things needed to be cleared before it gets accepted. In addition to the reviewers’ comments, authors may consider to response to the following observations:

1. I don’t think this statement is correct “Sleep duration is a reliable predictor of wellbeing [26] and affects QoL. Kripke et al. [27] observed 1.1 million American adults for six years and found that both extremely short (less than 4 hours) and extremely long (more than 8 hours) periods of sleep lead to elevated mortality.” I don’t think more than 8 hours sleep is considered as extremely long period of sleep, I see that the reference is from 2002, 20 years earlier than today!! National sleep foundation recommended hours for 18-64 is 7-9 hours. https://www.sleepfoundation.org/how-sleep-works/how-much-sleep-do-we-really-need

**RESPONSE:** We agree with the reviewer that labelling sleep duration as “extremely long” and “extremely short” is tricky because there is not a clear consensus on a such definition in literature. Thus we have toned down this statement to “too short” and “too long”. Instead of Kripke et al. 2002, we cite Cappuccio et al. 2010 in the current version as a more recent source that provides a systematic review and meta-analysis of 16 studies and 27 independent cohort samples. The definition of too short and too long sleep differs depending on the particular study: sleep is considered short if ≤ 4 h, ≤ 5 h, ≤ 6 h or < 7 h per night and long if ≥ 8 h, ≥ 9 h, ≥ 10 h, and ≥ 12 hours per night depending on a particular study.

Kripke, D., Garfinkel, L., Wingard, D., Klauber, M., & Marler, M. (2002). Mortality associated with sleep duration and insomnia. Primary Care Companion to the Journal of Clinical Psychiatry, 4(1), 34.

Cappuccio, F. P., D’Elia, L., Strazzullo, P., & Miller, M. A. (2010). Sleep duration and all-cause mortality: A systematic review and meta-analysis of prospective studies. Sleep, 33(5), 585–592. <https://doi.org/10.1093/sleep/33.5.585>

2. “Higher sleep quality” and “improvements” are these two are two different thing? Same applies for “Higher social jetlag levels” and “increases in social jetlag”

**RESPONSE:** Yes, “higher sleep quality” and “improvements in sleep” are two distinct things. “Higher sleep quality” and “higher social jetlag levels” refer to the differences between respondents, whereas “improvements” and “increases in social jetlag” relate to the differences within the same respondent in time. To clarify this, hypotheses were rephrased (pages 6 and 7).

3. “The average sleep duration in the CR is 7.5 hours (Table 1)” to me it information is not really helpful until it is presented by the age group.

**RESPONSE:** We agree with the reviewer that sleep duration varies between age groups. However, comparing average sleep duration in relation to age is not an aim of this article. We include this information to demonstrate that the average sleep duration of our sample is comparable to citizens in other countries.

To address the reviewer’s point, we are enclosing the table with average sleep duration concerning age categories (they were created using sextile function = creating six app. equally sized groups):

| Age category | N | Sleep duration |
| --- | --- | --- |
| up to 25 years old | 808 | 7.7h |
| 26-37 years old | 841 | 7.4h |
| 38-46 years old | 765 | 7.2h |
| 47-57 years old | 846 | 7.3h |
| 58-65 years old | 701 | 7.5h |
| 66 years and older | 562 | 7.8h |

4. Could you please consider explaining the H1 “Longer sleep duration and increases in sleep duration are related to higher levels of QoL.”. Firstly, please explain what do you mean by long sleep duration? How do you identify if someone is having longer explain? What is your cut-off? Secondly, “increases in sleep duration”, do you mean increase in sleep duration even if someone’s sleep duration fall within normal sleep hours. Please clarify.

**RESPONSE:** Firstly, in H1 we do not talk about “long sleep duration” *per se* but about relative time spent sleeping (less or more hours in comparison to other respondents), and about an increase in sleep length in time (i.e. increase in average sleeping time within the same respondent in time). There is no agreement in the literature on what can be considered “long sleep duration”, thus there is no precise cut-off to be made. Please note that we also do not make suggestions about what should be considered “normal”, “short” or “long” sleeping time. A short version of this clarification was also added on page 4.

Secondly, by “increases in sleep duration”, we mean an increase in hours spent sleeping between waves (e.e. within the same respondent in time). This is captured by the within-person variable. The length of sleep (average sleep hours) is captured by the between-person variable which is present in the model together with the within-person variable. To clarify this, hypotheses were rephrased (pages 6 and 7). We proceed like this because the focus of this article is on within (in time) and in between (between individuals) comparisons allowing us to identify the differences for the same respondent in time and in between respondents. This is also how are the results interpreted: e.g., in Table 2., there is a Model 5A with Happiness as a dependent variable and the results tell us that if sleep duration increases for one hour, happiness will decrease for -.56. In our interpretation, this means that on average longer sleep is related to higher levels of Happiness and therefore also QoL.

5. I am not sure what study design authors used in this study. Is it a cross-section at multiple points of time or was this study a cohort study? Please explain why “the retention rate of individuals was 20.6 %” is important if this is not a cohort study.

**RESPONSE:** The data we use were collected as a part of a longitudinal survey designed to follow the same sample on multiple occasions over time. More specifically, it is a household panel survey which has the main objective *“to provide a rich data resource to be used by a wide range of users for a broad set of purposes. The data structure is complex, involving interviews with each person in the household of each sample member at each wave, in addition to household-level information and, often, additional survey instruments such as self-completion questionnaires or health measurements” (Lynn, 2008: p. 2).* The Czech Household Panel Survey is rich in thematic focus and consists of answers from the adult population ranging from 18 years to 96 years of age and is also representative of the Czech population.

The design has advantages over cohort studies: it allows for analysis of unit-level change, and measures of stability within individuals and households, and also takes into consideration time-related characteristics while not having to wait for some sort of exposure to happen (e.g., reach a certain health outcome such as death). Also, relatively short follow-up periods such as three-time points in our case can be already interesting to look at.

Cohort studies can also be longitudinal, however, they focus on participants sharing common characteristics, usually focusing on a specific age group and the topics are more specified and also strictly age-related (Lynn, 2008).

To compare the two designs: while cohort studies aim to explain the effect of age on the life course, the purpose of panel studies is to monitor changes in time and also cross-sectional snapshots of the population (representative of the Czech population in our case).

The retention rate is an important indicator of data quality. We included this information in the study to increase transparency and also to depict the character of this longitudinal survey.

Lynn, P. (2009). *Methodology of longitudinal surveys*. John Wiley & Sons.

6. I understand this was secondary data analysis? Authors may considering writing more about the dataset including the design used and cite articles that has been published using this dataset.

**RESPONSE:** It was a secondary data analysis. We added a more detailed description of the survey and used the design in the current version of the manuscript. The complete official technical information of the survey is extensive (81 pages) and unsuitable to be thoroughly described in the article. Therefore, the technical information is also referred to in the first paragraph of the “Study design and participants” section in the “Data and methods chapter”.

The CHPS data are extensively used in articles published in prestigious journals. On page 8, we added references to some peer-reviewed articles that use the same data for analysing sleep patterns but also other topics. Mentioned studies are only a fraction of what is available.

7. Please consider describing PAPI questionnaire? Could you please also some words on the why sample is higher in 2020 (2,161) than earlier year 2019 (2,046)? …..since you stated “the sample size in 2020 was reduced for financial reasons.”

**RESPONSE:** PAPI stands for “Pen and Paper Interviewing” but after consideration, it was replaced in the text (page 8) by the more widely used “paper-and-pencil self-administered questionnaire” (SAQ).

We apologize for the confusing information. It was not the sample size that was reduced due to financial reasons but it was the length of the questionnaire. The sentence regarding sample size in 2020 was removed.

8. What was the age of the adult population?

**RESPONSE:** The population from which was the sample selected were citizens living in the Czech Republic that has reached 18 years or more. The original sample was selected randomly from the registry. The population age ranges from 18 up to 96 years: this information is also available in Table 1 in the manuscript and technical documentation (see also Kudrnáčová 2019).

9. Authors mentioned that “4,523 respondents in up to 2,155 households.” So I was wondering who did the study interviewed? And how it was determined? Is it both male and female in the same household? Please kindly describe.

**RESPONSE:** Agreed. We added more detailed information in the first paragraph of the “Study design and participants” section in the “Data and methods chapter” (pages 7 and 8): *“The original sampling frame from the very first data collection consisted of the Register of Census Districts and Buildings which had been transformed into an address database. Since the target population was the non-institutionalized population of the Czech Republic, all members of the sampled households were interviewed. In each of the following waves, the same members of the same households participating in the preceding wave were approached (e.g., in wave three in 2017, only participants from wave two were approached).”*

The survey’s target population comprises the non-institutionalized population of the Czech Republic irrespective of citizenship or ethnicity. A household was deemed eligible for the survey if it was able to be interviewed, i.e. at least some of its members spoke Czech or Slovak and interviewing was not prevented by their health condition. And if the household comprised both men and women in the household, then both were asked to participate in the survey.

10. Regards to all measures, do the measures comprise of multiple items or single item measure? I see that wellness has multiple items and I’d appreciate if you can provide the composite Cronbach alpha. Plus provide if this measure is validated scale.

**RESPONSE:** All measures of QoL and their properties are now explained in detail in the chapter “Data and methods” under the “Measures” title (pages 9-11).

To sum up, life satisfaction, happiness and subjective health are single-item measures, but they are widely used and validated measures; we cite selected studies using the same measures for their analytical purposes. Multiple-item measures are wellbeing (three items) and work stress(three items).

The composite Cronbach alpha of wellbeing is provided in the text for all waves of CHPS in which the scale is available (page 9): *“The resultant reliability estimates are acceptable (αt1 = .811; αt2 = .828; αt3 = .830; αt4 = .841; αt5 = .825).”* The three items provided are part of the validated WHO-5 Well-Being Index (WHO-5). It is also used as a three-item scale in the European Social Survey (ESS) questionnaire. An example of a study that includes the WHO-5 (Nordenmark, Vinberg & Strandh 2012) is cited in the paragraph describing this measure.

The three items that capture Work stress were inspired by the work stressors in European Working Conditions Surveys (EWCS) which are validated also as two and three-item proxies (Fransson et al., 2012; Karasek et al., 2007) and frequently used in peer-reviewed articles (e.g., Rigó et. al, 2021).

Fransson, E. I., Nyberg, S. T., Heikkilä, K., Alfredsson, L., Bacquer, D. D., Batty, G. D., Bonenfant, S., Casini, A., Clays, E., Goldberg, M., Kittel, F., Koskenvuo, M., Knutsson, A., Leineweber, C., Magnusson Hanson, L. L., Nordin, M., Singh-Manoux, A., Suominen, S., Vahtera, J., … Kivimäki, M. (2012). Comparison of alternative versions of the job demand-control scales in 17 European cohort studies: The IPD-Work consortium. *BMC Public Health*, *12*(1). https://doi.org/10.1186/1471-2458-12-62

Karasek, R., Choi, B., Ostergren, P.-O., Ferrario, M., & de Smet, P. (2007). Testing Two Methods to Create Comparable Scale Scores between the Job Content Questionnaire (JCQ) and JCQ-Like Questionnaires in the European JACE Study. In *International Journal of Behavioral Medicine Copyright C* (Vol. 14, Issue 4). Lawrence Erlbaum Associates, Inc.

Nordenmark, M., Vinberg, S., & Strandh, M. (2012). Job control and demands, work-life balance and wellbeing among self-employed men and women in Europe. Vulnerable Groups & Inclusion, 3(1), 18896. https://doi.org/10.3402/vgi.v3i0.18896

Rigó, M., Dragano, N., Wahrendorf, M., Siegrist, J., & Lunau, T. (2021). Work stress on rise? Comparative analysis of trends in work stressors using the European working conditions survey. *International Archives of Occupational and Environmental Health*, *94*(3), 459–474. https://doi.org/10.1007/s00420-020-01593-8

11. Also please consider mentioning the interpretation of the scale, i.e., what is the higher and lower score mean of some measures?

**RESPONSE:** The wellbeing scale and its composition are now explained in detail in the chapter “Data and methods” under the “Measures” title (page 9):

*“Wellbeing was calculated as an average of three items to measure how often over the last two weeks respondents “have been cheerful and in good spirits”; “have felt calm and relaxed”; “have been active and vigorous”. The six response options were “at no time”, “some of the time”, “less than half of the time”, “more than half of the time”, “most of the time”, “all of the time”. The resultant reliability estimates are acceptable (αt1 = .811; αt2 = .828 αt3 = .830; αt4 = .841 αt5 = .825). The same items were measured during two out of three analysed years of data collection in 2018 and 2019 and have also been used to measure wellbeing in other studies (Nordenmark et al., 2012).*

For clarification and readability improvement, we added an explanation of what each score means (page 9). Information regarding the minimum and maximum of the scale is also included in Table 1.

Nordenmark M, Vinberg S, Strandh M. Job control and demands, work-life balance and wellbeing among self-employed men and women in Europe. Vulnerable Groups Incl. 2012;3(1):18896.

12. Authors did not mentioned about the normality assumptions of continuous variables? I see from table 1, max age is 94 years. I don’t know if it should be considered as outlier. Plus, SD is 22.51. Also the sleep duration is max 13.48 (hours I believe). I m sure if that should also be considered as outliers.

**RESPONSE:** We are enclosing the histogram of age and sleep duration and the dataset along with syntax with our analyses was made available as part of the Supporting information. Based on this visual inspection, we do not consider the provided maximums as outliers. Moreover, sleep duration was already cleansed of outliers (less than 3.5 and more than 14 hours of sleep cases were omitted from the analyses).

Even though age is not normally distributed, it is not an issue for our analyses. The condition for Multilevel mixed-effects linear regression with repeated measurements is the random intercept and random slope are assumed to follow a bivariate normal distribution. Also, given the size of the sample, a small deviation from normality is considered unproblematic (Ghasemi and Zahediasl, 2012).

Ghasemi, A., & Zahediasl, S. (2012). Normality tests for statistical analysis: A guide for non-statisticians. International Journal of Endocrinology and Metabolism, 10(2), 486–489. https://doi.org/10.5812/ijem.3505

13. Presenting the table by data points (2018, 2019, and 2020) would be useful to see the mean changes over time. Seeing the wave 4-5-6 reduces the readability of the paper.

**RESPONSE:** We thank the reviewer for this comment. We reduced the frequency of wave numbers used and replaced them with years instead. We agree it improves the readability of the paper.

The mean changes are a central point to our analyses as presented in Table 2 and Table 3. Table 1 only serves as an introduction to the data and respondents’ characteristics.

14. Table 1, please kindly explain the meaning of mean education 1.18. Is this the years of education completed? Also clarify the word “maturity exam” for increasing the readability for the international audience.

**RESPONSE:** We understand the confusion regarding education variables. Table 1 values are calculated as an average of Czech educational levels (1- basic and secondary vocational, 2 - secondary with maturita, 3 - tertiary education) in CHPS waves used in the analyses. As suggested, we changed the wording of maturita exam to “secondary education with maturita” to increase the readability. Hopefully, it is now clearer that “secondary education with maturita” is a higher level of education than “basic and secondary vocational” but less than “tertiary education”.

15. Please discuss the limitations of the item scale used all most of the measure since I believe it was measures by 1 item scoring. Plus I saw some scale asked specific time duration while some ae general subjective response without time boundness. For example, sleep duration and some other have not time restricted response while welling was over the last two weeks.

**RESPONSE:** We added clarification of scoring of the Wellbeing measurement which is a validated scale (page 9). Some aspects of quality of life are measured with one-item questions but we acknowledge this fact in the chapter “Data and methods” under the “Measures” for each measure. These measures are also widely used which we demonstrate by citing selected articles using the same measures. As for the sleep variables, only sleep duration and social jetlag are computed as time variables but they were transformed to integer values (see Table 1 for minimum and maximum values of both). However, this treatment is in line with previous research and they are computed based on Munich Chronotype Questionnaire (MCTQ; WEP, 2020) as it is also described in the manuscript. We also recognize the limitations of such subjective measures in the “Limitations section” (pages 18 and 19): *“Fourth, the sleep indicators are self-reported and therefore have limitations despite self-reported measures being similarly reliable predictors (Wunsch et al., 2021). Ideally, the measures would be collected in a medical lab or via mobile devices to aid in cross validating our results with more objective methods of measurement.”*

WEP. MCTQ [Internet]. 2020 [cited 2020 Jan 24]. Available from: https://www.thewep.org/documentations/mctq

Wunsch K, Nigg CR, Weyland S, Jekauc D, Niessner C, Burchartz A, et al. The relationship of self-reported and device-based measures of physical activity and health-related quality of life in adolescents. Health Qual Life Outcomes [Internet]. 2021;19(1):1–10. Available from: https://doi.org/10.1186/s12955-021-01682-3

16. Table 2, please explain what does the numbers mean in the parenthesis. For example, 0.06 (-.14- .26)…..is this CI or SD or SEM. I suggest to write 95%CI.

**RESPONSE:** We thank the reviewer for the comment regarding the presentation of confidence intervals and we are sorry for the confusion. We have clarified CI in the current version of our manuscript in the notes of the tables.

17. What was your random effect variable in table 2 and table 3.

**RESPONSE:** The random effect was in both tables for households, which is specified as a random intercept term which varies the household’s intercept around the estimated (mean) constant term. Another random effect variable was the time which varied based on waves (2018, 2019 and 2020). The updated version of the manuscript is accompanied with the dataset and syntax used for analyses in Supporting information.

18. Table 2 and Table 3, please place the household income in USD for international audience. Or may be include both, local and international currencies.

**RESPONSE:** Agreed. In the current version of the manuscript, we describe the categories of income in two currencies – CZK and USD (footnote on page 10). We also added information regarding the average monthly household income in the Czech Republic.

19. I’d like to know more on how models were built. I see that all the independent variables were included in the final model. Also would you please provide the model fit statistics.

**RESPONSE:** The models were built using mixed, multilevel repeated measurement models with random intercepts for individuals, households and a random slope for time.

We explain how the models were built in detail on page 12: *“We started with null models which incorporated the dependent variables without predictors to capture the variance of the dependent variables. Next, we examined the longitudinal effects of the three tested facets of sleeping hygiene on the five measures of QoL by adding sleep duration, sleep quality and social jetlag variables and interaction terms for time and sleeping variables (Models 1A–5A). In the final step, Models 1B–5B decomposed the effects of sleeping on within-person and between-person effects. We then evaluated the model fits according to the general principle that models with lower deviance and AIC values than the null model are considered better fitting models (Kreft and Leeuw, 1998).”*

We now include our dataset with syntax that we used for analysis as Supplementary Information for this article.

Kreft IG, Leeuw J de. Introducing Multilevel Modeling. SAGE Publications, Ltd; 1998. 160 p.

20. Could you please put the null model table as supplement.

**RESPONSE:** Agreed. A table including information regarding null models was added as S2 Table 1.

Review Comments to the Author

Please use the space provided to explain your answers to the questions above. You may also include additional comments for the author, including concerns about dual publication, research ethics, or publication ethics. (Please upload your review as an attachment if it exceeds 20,000 characters)

Reviewer #1: The manuscript Better Sleep, Better Life? Testing the Role of Sleep in Quality of Life has as main goal to establish the relationship between sleep or some of its features with quality of life. I consider that they have a good sample size, the questionnaire about QoL is very well chosen and explained.
However 1.the counterpart , the sleep questionnaires allow the participants to be very subjective, that could be observed in some questions that are al least inconsistent (as an example, the quality of sleep has a negative correlation with work stress in retired subjects, and there are other items alike).

**RESPONSE:** The questions as they are posed in the questionnaire are of a subjective nature. We worked with what we had at our disposal which was data for secondary analysis. Unfortunately, collecting objective measures with smart devices or in the sleep lab, was not part of the data collection even though it would have been ideal. We acknowledge this measurement limit in the “Limitations” section. However, the Munich Chronotype Questionnaire (MCTQ) is a valid and widely used tool to measure the sleep components, and also if the data would have been collected using smart devices or in a sleep lab, there would be a limited number of respondents instead of a representative sample.

2. Sleep quality classified only by the answers without some complementary information is hard to interpret

**RESPONSE:** Generally, single items are widely used in social research, especially in large surveys and they prove to be reliable and valid tools when correlated with multi-item measures (Diener, 1984). Specifically, the single-item measure of sleep quality has been standardly used in several studies (e.g., Carney et al., 2012, Foley et al., 2004). Although more objective measures of sleep quality would have been preferable the subjective measures are standard in social science research. Previous studies comparing these measures found that results obtained using objective and subjective measures differ a bit, but they stress that subjective measures are important and correlate with diseases of ageing and mortality (Landry et al, 2015). We acknowledge the potential limits of our sleep measures in the “Limitations” section (pages 18 and 19).

Carney, C. E., Buysse, D. J., Ancoli-Israel, S., Edinger, J. D., Krystal, A. D., Lichstein, K. L., & Morin, C. M. (2012). The consensus sleep diary: Standardizing prospective sleep self-monitoring. In *Sleep* (Vol. 35, Issue 2, pp. 287–302). <https://doi.org/10.5665/sleep.1642>

Diener, E. (1984). Subjective well-being. *Psychological Bulletin*, *95*(3), 542–575. https://doi.org/10.1037/0033-2909.95.3.542

Foley, D., Ancoli-Israel, S., Britz, P., & Walsh, J. (2004). Sleep disturbances and chronic disease in older adults: Results of the 2003 National Sleep Foundation Sleep in America Survey. *Journal of Psychosomatic Research*, *56*(5), 497–502. <https://doi.org/10.1016/j.jpsychores.2004.02.010>

Landry, G. J., Best, J. R., & Liu-Ambrose, T. (2015). Measuring sleep quality in older adults: A comparison using subjective and objective methods. *Frontiers in Aging Neuroscience*, *7*(SEP). https://doi.org/10.3389/fnagi.2015.00166

3.Therefore some results that could be more objectives like sleep duration (time to sleep and wake up) or the social jet lag showed results similar to the literature and performed some of them with more more objective measures.

**RESPONSE:** For the computation of sleep duration, we are using the average sleep duration during the week which is computed in line with the Munich Chronotype Questionnaire (MCTQ) which is a valid and widely used tool to measure the sleep components (Sládek et al., 2020; Wittman et al., 2006).

We acknowledge this limitation on pages 18 and 19 and agree with the reviewer that more objective measures of sleep indicators would be great. However, they are not available to us and we acknowledge their limits. However, these measures are standardly used in other studies (Borisenkov et al., 2019; Roenneberg et al., 2003; Sládek et al., 2020; Wittman et al., 2006) and have been successfully validated against biochemical biomarkers (Roenneberg et al., 2019).

Borisenkov, M. F., Vetosheva, V. I., Kuznetsova, Y. S., Khodyrev, G. N., Shikhova, A. v., Popov, S. v., Pechеrkina, A. A., Dorogina, O. I., & Symaniuk, E. E. (2019). Chronotype, social jetlag, and time perspective. *Chronobiology International*, *36*(12), 1772–1781. https://doi.org/10.1080/07420528.2019.1683858

Roenneberg, T., Wirz-Justice, A., & Merrow, M. (2003). Life between clocks: Daily temporal patterns of human chronotypes. *Journal of Biological Rhythms*, *18*(1), 80–90. <https://doi.org/10.1177/0748730402239679>

Roenneberg, T., Pilz, L. K., Zerbini, G., & Winnebeck, E. C. (2019). Chronotype and social jetlag: A (self-) critical review. *Biology*, *8*(3), 54. https://doi.org/10.3390/biology8030054

Sládek M, Kudrnáčová Röschová M, Adámková V, Hamplová D, Sumová A. Chronotype assessment via a large scale socio-demographic survey favours yearlong Standard time over Daylight Saving Time in central Europe. *Sci Rep*. 2020;10(1):1–18.

Wittmann, M., Dinich, J., Merrow, M., & Roenneberg, T. (2006). Social jetlag: Misalignment of biological and social time. *Chronobiology International*, *23*(1–2), 497–509. <https://doi.org/10.1080/07420520500545979>

4. I think that to compare individuals and households did not give a particular contribution to the discussion.

**RESPONSE:** We agree that comparing individuals and households would not contribute to the discussion. We actually do not compare individuals and households. Households are the primary sampling unit in the survey and as such, they are important to be controlled for in analyses because there is a reason to believe that individuals from the same household might have similar behaviour patterns. Without controlling for household membership we may get biased results (Hox 2002).

Hox, J. J. (2002). *Multilevel Analysis: Techniques and Applications*. Mahwah, NJ: Lawrence Erlbaum Associates Publishers. https://doi.org/10.4324/9781410604118

5. We know that when the projects were designed, we did not know about the pandemic. But, the fact that the last year was an especial time of the life, it might not reflect the regular behavior of the participants and could introduce a bias to the final results. With this scenario to choose one year before (2016) for example would have been better. It could be possible for increasing the interest of the research to create extreme groups (in sleep or QoL fields) and study if they have a different behavior. In summary, the more important result that it appears to be that sleep quality is associated positively with QoL is based on a soft base that support similar results from other studies but does not contribute to deepen knowledge about the closest relationship between life asleep and life awake.

**RESPONSE:** Unfortunately, sleep variables were included in the survey only in 2018, 2019 and 2020. In other words, we used all the waves that were available and could not choose which year would be more or less suitable. Our central research question is how changes in QoL are related to changes in sleeping habits. We do not investigate the sources of these changes. We, however, agree that this is also an important aspect worth examining and hopefully someone will investigate this in the future. This intriguing suggestion for future research was added to the final section of our manuscript (page 20).

We believe that our manuscript does contribute to the ongoing discussion. 1) In contrast to the previous research, we test which sleeps aspect out of the three (quantity, quality, social jetlag) has the strongest effect on QoL that we measure by five parameters capturing all areas of one’s life according to integrative quality-of-life (IQOL) theory. This has never been done before. 2) Longitudinal studies on representative samples are very rare. Our study is longitudinal and thus allowing us to interpret both between-person and within-person differences. 3) There is a shortage of studies linking sleep and QoL that originate outside “western democracies”.

Reviewer #2: This study is imperative because it examined the impact of sleep on QoL. I would definitely recommend this paper for publication. The methodology of the study is solid and convincing, and the study findings enable further research on multidimensional QoL and sleeping patterns. I only have a few comments that could perhaps improve this write-up: 
1. I would like to suggest if the author could condense the introduction section. I find the introduction a bit lengthy and there is a mix of discussion in the introduction section. The summary of the previous research studies definitely helps readers to understand the background and loops of the previous studies.

**RESPONSE:** We have made changes to increase readability according to the reviewer’s suggestions. The introductory section has been shortened and rewritten to be focused on sleeping patterns and their effect on the quality of life.

2. The authors highlighted the hypothesis of the study, but I would recommend a clear study objective in the introduction section.

**RESPONSE:** Agreed. The objective is stated in the very last sentence in the introduction (page 2): *“Using three waves of the Czech Household Panel Study data, the present study contributes to the literature by examining the effect of sleep duration, sleep quality and social jetlag on five QoL indicators and exploring the trends in time.”*

3. Some information in the method is unnecessary, i.e., "Case selection" could perhaps be written in the introduction section or maybe the discussion section, as the authors were comparing the Czech people with the other European nations. Or maybe they simplified the case selection of respondents that were included in this study.

**RESPONSE:** Agreed. We have moved the part emphasizing the comparability of the Czech case to people from other countries and that the results can be, therefore, reasonably generalized to other countries to the “Discussion” (pages 16 and 17).

4. Please describe clearly 'PAPI' when first introduced.

**RESPONSE:** PAPI stands for “Pen and Paper Interviewing” but after consideration, it was replaced in the text by the more commonly used “paper-and-pencil self-administered questionnaire” (SAQ) term. We thank the reviewer for this comment which helped to clarify the used term.

5. I would like to know exactly what tools were used to measure QoL in this study. Are the QoL tools newly developed? or adopted from other established QoL tools? Are the tools used specifically able to measure QoL for sleeping patterns or certain age groups? The authors need to elaborate on the tools used, especially on their suitability for certain age groups. older than adults?

**RESPONSE:** The tools were not developed by the authors. All the measurement tools used in our analyses are based on previously validated and widely used tools. They are based on the concept that mostly originates in sociological literature. In the current version, we put more references and information regarding the origin of the tools to increase their credibility.

In general, we base our analysis on the subjective component of all-embracing integrative quality-of-life (IQOL) theory (Fig 1) which is in-depth elaborated on in the “Quality of life definition” chapter and in our approach, we use life satisfaction, wellbeing, happiness, subjective health and work stress as indicators of QoL.

We understand the reviewer’s concern about differences in the measurement of QoL between various age groups. All the tools used in this study were developed to capture variables that are valid for an adult population. There is no particular reason to believe that validity of the variables is dependent on the respondent’s age. It is important to note, that we do not compare results between age groups. We analyse the effect of sleep on the QoL of the general population and as such, we need to use the same measurement tools for all respondents.

6. Need more inputs on the discussion, especially comparing QoL and sleeping with other countries, authors' tools vs other QoL tools that measure QoL in sleeping.

**RESPONSE:** In the discussion of the current manuscript we compare the Czech case with other European countries in terms of various aspects of the QoL. We also rewrote the “Data and methods” section to emphasize that our variables were measured by standard items that are commonly used in sociological research. We supported that by adding references to such studies. More specifically, the central sleep variables were collected by using Munich Chronotype Questionnaire (MCTQ). It contains validated and widely used measures (see “Data and methods” section) for each sleep variable. Another tool, that is used, is the Pittsburgh Sleep Quality Index (PSQI). Both MCTQ and PSQI are subjective since they are self-reported by the respondents. We are aware that using objective measures instead of subjective tools, or ideally a combination of both would be an asset. Therefore, we acknowledge this limitation of our tools in the limitation section of our manuscript. Ideally, we would also have measurements taken in the sleep laboratories or by smart devices. This was not possible with our data sets. However, the data we use has other advantages such as longitudinal structure, a high number of respondents, a combination of several sleep measures as well as QoL indicators in the same questionnaire, which taken together have the potential to make an important contribution to the literature.

7. Lastly, did the authors control for confounders such as those with mental health problems in the study or exclude those who are clinically diagnosed with sleeping problems in this study (if any).

**RESPONSE:** In Table 2 and Table 3 are included all the variables entered in our models. Also, to be completely transparent, we included the minimal dataset and syntax used for analyses as part of the Supporting information. Specifically, mental health problems were not controlled for because the study aimed to examine the effects of selected sleep variables on QoL, so we included only sociodemographic variables and sleep variables as confounders. Moreover, some aspects of QoL could be considered as partially capturing mental health state (especially subjective health) and it would have been, therefore, problematic to include mental health problems.

The respondents were recruited under the condition that the interviewing was not prevented by their health condition. Unfortunately, it is not possible to identify respondents clinically diagnosed with sleeping problems and control for this in our analyses.
