# Supplementary material for: Better sleep, better life? testing the role of sleep on quality of life
Source: PLoS One. 2023 Mar 15;18(3):e0282085. doi: 10.1371/journal.pone.0282085 (PMC10016705; doi:10.1371/journal.pone.0282085)
Supplement: Attachment — Submitted filename: Response to reviewers.docx [file pone.0282085.s004.docx]

Journal Requirements:

Please review your reference list to ensure that it is complete and correct. If you have cited papers that have been retracted, please include the rationale for doing so in the manuscript text, or remove these references and replace them with relevant current references. Any changes to the reference list should be mentioned in the rebuttal letter that accompanies your revised manuscript. If you need to cite a retracted article, indicate the article’s retracted status in the References list and also include a citation and full reference for the retraction notice.

**RESPONSE:** One typo and several duplicates were identified in the reference list. The reference is complete and correct. As far as the authors are concerned, no retracted articles were cited. Due to the nature of the changes, they were not made via the “track change” function. After applying the formal changes to the Manuscript, this updated version was used as a baseline for the new version of “Manuscript” as well as “Revised manuscript with track changes”.

1. Improve the quality of the table. For example , is better to do different tables for socio-economic variables, and other tables for the variables of the study. Besides , is cleaner if the intraindividual and inter-individual data could be seen apart.

**RESPONSE:** We understand the reviewer’s concern about the readability of the tables. Table 2 and Table 3 are created in compliance with the existing recommendations on how to report multi-level repeated measure designs (e.g., Quené and van den Bergh, 2004), and they are typically reported as one compact table to correspond to how they were computed (see S1_replication_syntax that we enclosed along with the data). To address the reviewer’s comment, we decided to improve the tables by grouping inter-individual variables and inter-individual variables, and by adding an extra column with headings (e.g., socio-demographic variables, interaction effects, sleep variables) to the left side of Table 2 and Table 3 to improve structure of the Tables, which in turn increased readability for the readers.

Quené, H., & Van den Bergh, H. (2004). On multi-level modeling of data from repeated measures designs: A tutorial. Speech communication, 43(1-2), 103-121.

2. It is important to reinforce that the study was done during COVID pandemia and might not be representative of the behavior in normal conditions.

**RESPONSE:** This is reflected in the Limitations section on page 17 but we agree with the reviewer that it needs to be emphasized even more thus we added an extra sentence to clarify this: *“Fifth, even though data were collected on regular days, the final wave partially captured the experience of the pandemic in the spring of 2020,* ***and this study, therefore, might not be representative of the behavior under normal circumstances****. However, data collection occurred during periods of eased restrictions and likely did not affect the generalizability of the results.”*
